# Ocular tuberculosis masquerading as atypical ocular toxoplasmosis

**DOI:** 10.3205/oc000227

**Published:** 2023-09-29

**Authors:** Albert John Bromeo, Sweet Jorlene Lerit, Cheryl Arcinue

**Affiliations:** 1 Asian Eye Institute, Makati, Philippines; 2 Department of Ophthalmology and Visual Sciences, Philippine General Hospital, University of the Philippines, Manila, Philippines

**Keywords:** tuberculosis, toxoplasmosis, uveitis, retinitis, masquerade syndrome

## Abstract

Ocular tuberculosis is a great mimicker of various uveitis entities. We present a case of a 29-year-old male who came in with blurring of vision and floaters in the left eye. On examination, the left eye had anterior chamber cells and vitritis associated with retinitis. He had no other symptoms. The initial presentation was consistent with ocular toxoplasmosis, and he was started on oral sulfamethoxazole-trimethoprim and showed a good response to the treatment. However, work-up revealed negative toxoplasma antibody titers but a positive *M. tuberculosis* interferon-gamma release assay test and Mantoux test, making the diagnosis of ocular tuberculosis more likely. The patient was shifted to antituberculous therapy, which eventually resulted in the resolution of the inflammation with a recovery of the visual acuity. The diagnosis of ocular tuberculosis requires a detailed medical history as well as microbiologic and immunologic studies. A high index of suspicion by the treating ophthalmologist is necessary to reveal the diagnosis.

## Introduction

Tuberculosis (TB) is an infection caused by the acid-fast bacillus *Mycobacterium tuberculosis*. Ocular tuberculosis is an extrapulmonary mycobacterial infection with a myriad of manifestations. While considered rare in developed countries, the condition is quite common in the Philippines with an estimated prevalence of 6.8% [1]. The diagnosis of ocular TB can be difficult as it can present with any form of intraocular inflammation. The various presentations of the disease are due to the location of infection, the host response, and the virulence of the bacteria. Thus, clinicians need to maintain a high index of suspicion as the disease may mimic autoimmune inflammatory conditions, tumors, and even other infections.

We present the case of a peculiar presentation of ocular TB which initially masqueraded as ocular toxoplasmosis, another common intraocular infection.

## Case description

**Figure 1:**
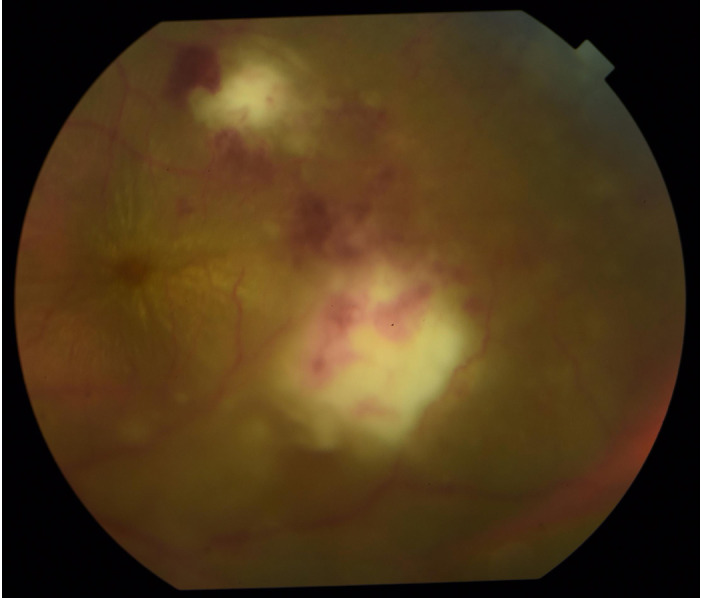
Fundus photograph of the left eye showing foci of retinitis at the temporal aspect of the macula associated with exudates arranged in a macular star

A 29-year-old male presented with a 2 month history of blurring of vision with floaters on the left eye. He had no other symptoms and no previously diagnosed comorbid illnesses. On ophthalmological examination, the left eye had a best corrected visual acuity (BCVA) of 20/30-2. Biomicroscopy identified 2+ cells with 2+ flare in the anterior chamber. There were no keratic precipitates. The intraocular pressure was 18 mmHg. Funduscopy showed vitritis with snowballs, and retinitis associated with hemorrhages at the distal ends of the temporal arcades (Figure 1 (Fig. 1)). There were associated retinal exudates arranged in a macular star configuration. The right eye was essentially normal. 

**Figure 2:**
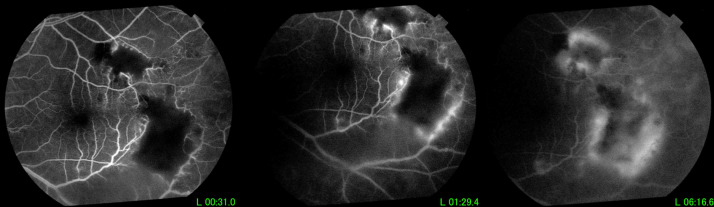
Fluorescein angiogram showing blocked hypofluorescence from retinal edema adjacent to areas of retinal vasculitis

**Figure 3:**
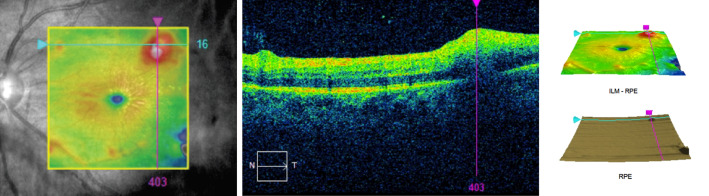
Optical coherence tomography scan through the lesion showing hyperreflectivity of the inner retinal layers with posterior shadowing, consistent with retinitis

The fluorescein angiogram showed areas of retinal vasculitis along the distal ends of the temporal arcades near the peripheral areas of retinitis. The foci of retinitis appeared as blocked hypofluorescent lesions (Figure 2 (Fig. 2)). Optical coherence tomography scan through the lesion showed hyperreflectivity of the inner retinal layers with posterior shadowing (Figure 3 (Fig. 3)), consistent with retinitis. There were no abnormalities of the retinal pigment epithelium and choroid.

Due to the presentation of vitritis associated with focal foci of retinitis, an initial working hypothesis of ocular toxoplasmosis was made. However, presentation was noted to be similar to atypical forms of toxoplasmosis, presenting with features such as multifocal lesions, hemorrhages, macular star, and absence of chorioretinal scars. An infectious panel, including testing for human immunodeficiency virus 1 and 2 (HIV-1 and HIV-2), was ordered. The patient was started on empiric antibiotic therapy consisting of a course of oral trimethoprim-sulfamethoxazole while awaiting the results of the systemic work-up. 

**Figure 4:**
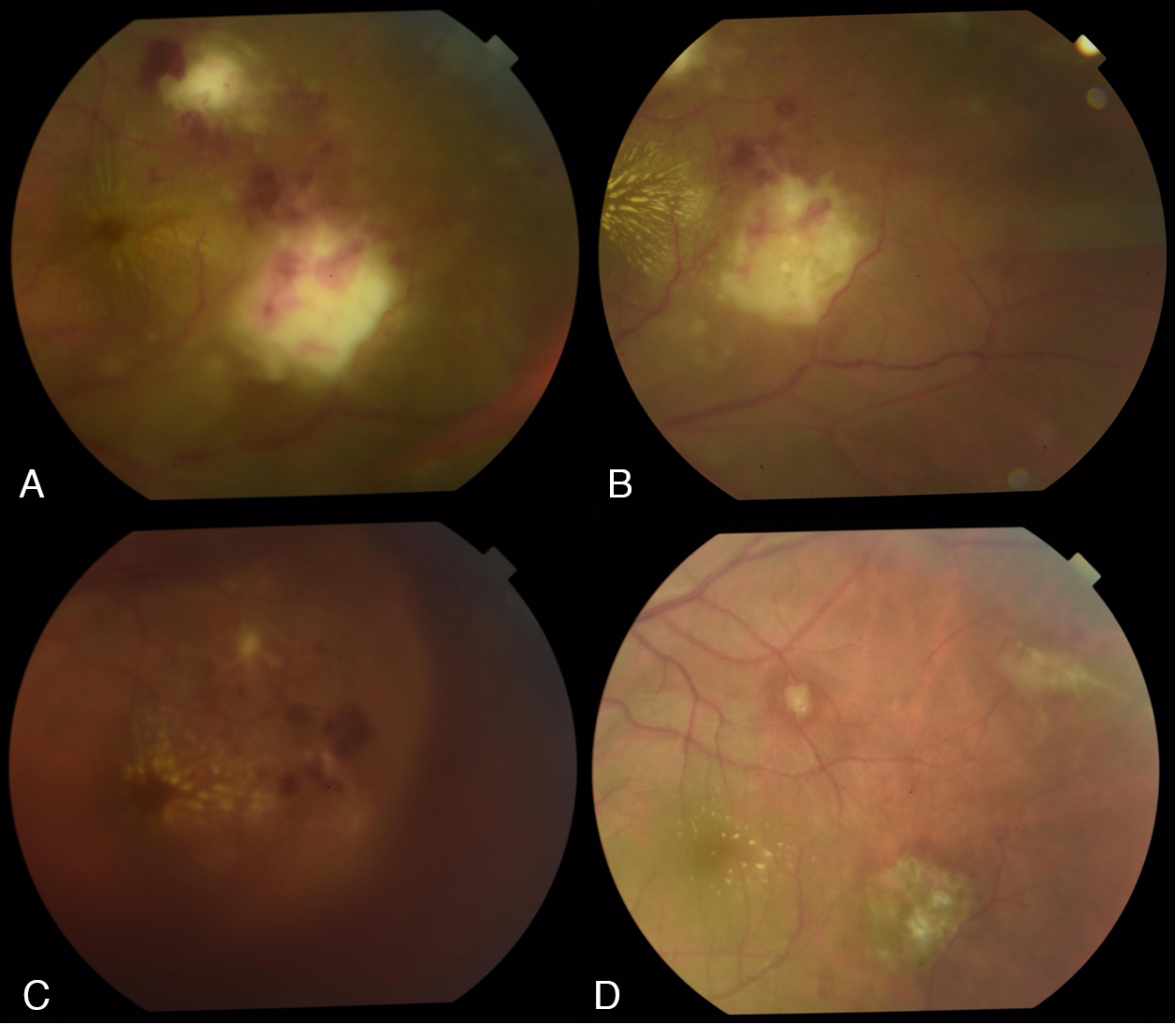
Serial fundus photographs showing the response of ocular inflammation with the various treatment regimens started. The fundus at (A) baseline, (B) after 1 week of antitoxoplasmic therapy, (C) after 1 month of antituberculous therapy, and (D) after completion of antituberculous therapy

The patient returned after a week with a good response to the treatment. Compared with baseline (Figure 4A (Fig. 4)), there were decreased vitritis, retinitis, and hemorrhages (Figure 4B (Fig. 4)) with accompanying improvement in BCVA to 20/25. However, the results of his work-up were not yet available. Because of ongoing inflammation, he was advised to continue the course of oral trimethoprim-sulfamethoxazole.

The patient returned after two weeks with the results of the infectious panel. Surprisingly, he had negative *Toxoplasma* IgG and IgM antibody titers. There was a positive *M. tuberculosis* interferon-gamma release assay (QuantiFERON) test. In addition, there was a positive reaction in the tuberculin purified protein derivative (PPD) skin test with an induration of 12 mm. The rest of the infectious panel, including tests for syphilis and HIV, was negative. The chest x-ray was essentially normal. Investigations for cytomegalovirus (CMV), herpes simplex virus (HSV), and varicella-zoster virus (VZV) were not done. A diagnosis of ocular tuberculosis was made.

The patient was shifted to anti-tuberculous therapy (ATT) consisting of an initial two-month four-drug regimen consisting of isoniazid 5 mg/kg daily, rifampicin 10 mg/kg daily, pyrazinamide 20 mg/kg daily, and ethambutol 15 mg/kg daily, followed by a four-month two-drug regimen consisting of isoniazid and rifampicin.

Within the first month of shifting to ATT, the patient developed vitreous hemorrhage (Figure 4C (Fig. 4)), which caused deterioration of his BCVA to 20/70-1. The condition seemed to have converted to an Eales disease-like presentation, which can be associated with *Mycobacterium tuberculosis* infection [2], [3]. ATT was continued, and he was advised to maintain an upright head position. Monthly monitoring during the course of treatment showed a slow but gradual resolution of the vitreous hemorrhage with no reactivation of posterior segment inflammation. A consideration was made regarding the initiation of corticosteroids. However, since the patient was responding well to ATT alone, including control of inflammation and hemorrhage as well as maintenance of good visual outcome, corticosteroids were not given. By the end of his six month course of ATT, there was a complete resolution of both ocular inflammation and vitreous hemorrhage (Figure 4D (Fig. 4)) and BCVA returned to 20/20.

## Discussion

*Mycobacterium tuberculosis* affects the eye either through a direct invasion of the microorganism or as an immune-mediated hypersensitivity reaction to the microorganism originating from a focus elsewhere in the body. Ocular TB may occur as an isolated finding with no clinical or laboratory evidence of pulmonary infection. The manifestations of ocular TB are diverse. It usually presents as an inflammatory disease and can manifest as an anterior, intermediate, or posterior uveitis, or panuveitis. The classic presentation of ocular TB occurring as posterior uveitis includes choroiditis with choroidal tubercles [4]. These appear as small, white to yellow, subretinal nodules with indistinct borders. In TB usually the choroid is affected due to its high blood flow and the affinity of *M. tuberculosis* for sites with oxygen-rich tissue. Other common presentations of ocular TB in the posterior pole include choroidal tuberculomas, serpiginous-like choroiditis, neuroretinitis, and vitreous hemorrhage (linked to Eales disease). Isolated retinal involvement is rare [4].

Ocular TB can masquerade as intraocular malignancy or other inflammatory conditions. Ocular toxoplasmosis is an inflammatory disease caused by the protozoan parasite *Toxoplasma gondii*. It usually presents as a focal necrotizing retinochoroiditis with an overlying area of vitritis, similar to the initial presentation of our case [5]. The diagnosis of ocular toxoplasmosis is often based on characteristic clinical findings. However, there are atypical presentations that may be missed by clinicians, resulting in a delay in diagnosis and treatment. Atypical toxoplasmosis may present with large, multiple, and/or bilateral lesions. In addition, other manifestations include punctate outer retinal toxoplasmosis, retinal vascular occlusions, retinal vasculitis, neuroretinitis, pigmentary retinopathy, optic neuropathy, and scleritis. Atypical presentations usually present in immunocompromised patients [5]. Diagnosis of ocular toxoplasmosis in atypical forms may be done through several methods, including testing of intraocular fluids and tissue by antibody tests, PCR, histopathologic examination, or a combination of these [6].

While ocular TB can also present with retinitis, isolated retinal inflammation has scarcely been reported compared to choroidal inflammation [7]. In addition, the inflammation in the present case initially responded to antibiotics for toxoplasmosis. Diagnostic consideration or a high clinical index of suspicion against masquerading ocular TB is required, particularly in areas of high incidence and prevalence such as the Philippines.

The diagnosis of ocular tuberculosis remains an arduous task for the ophthalmologist. Currently, there are no established diagnostic criteria for ocular TB. Corroborative evidence from a number of diagnostic tests is often needed, including tuberculin skin test (TST) or Mantuox test, interferon-gamma release assay (IGRA), and various other serodiagnoses [7], [8]. In our case, the diagnosis of ocular TB was confirmed through a positive IGRA and positive tuberculin PPD test. The current United States Centers for Disease Control (CDC) guidelines state that IGRAs may be used in all situations where the PPD test is also used, while United Kingdom guidelines suggest that IGRAs should only be used to confirm a positive PPD test [8], [9]. Recent studies indicate that the interpretation of IGRAs together with PPD results in a higher sensitivity for the diagnosis of ocular TB [7], [10]. These tests have been shown to be particularly useful in countries with a high burden of disease in their population, such as the Philippines [1]. While the gold standard remains culture identification of *Mycobacterium*, there is often relative paucity of the pathogen in the lesions [6]. A definitive diagnosis is often difficult to ascertain and the diagnosis is presumptive in most cases [7], [11].

Ophthalmologists need to maintain a high index of suspicion for ocular TB. TB should be excluded in all cases of posterior uveitis, particularly in countries with a high prevalence rate of tuberculosis.

## Conclusion

Ocular tuberculosis is an extrapulmonary form of *Mycobacterium tuberculosis* infection. It may occur with or without associated pulmonary infection. It can present as an anterior, intermediate, or posterior uveitis, or panuveitis. It can mimic the presentation of other inflammatory diseases, intraocular tumors, and other intraocular infections. A high clinical index of suspicion is required in the approach of such cases, particularly in areas of high prevalence of tuberculosis. Corroborative evidence of clinical findings and diagnostic work-up is necessary to reveal the diagnosis.

## Notes

### Authorship

All authors ensure that they have made a substantial contribution to the article and that they are in agreement with the form and contents of the manuscript.

### Ethics statement and informed consent

The case report is a minimal risk study which was conducted in full compliance with the principles of the Declaration of Helsinki and Good Clinical Practice. All identifying patient information was kept confidential. Informed consent was obtained from the family of the patient.

### Competing interests

The authors declare that they have no competing interests.
